# Supplementary material for: Hotspot areas of risky sexual behaviour and associated factors in Ethiopia: Further spatial and mixed effect analysis of Ethiopian demographic health survey
Source: PLoS One. 2024 May 31;19(5):e0303574. doi: 10.1371/journal.pone.0303574 (PMC11142568; doi:10.1371/journal.pone.0303574)
Supplement: Attachment — Submitted filename: Review comments ton Authors.docx [file pone.0303574.s002.docx]

**Comments to author(s)**

**Manuscript Title**: ***Hotspot areas of risky sexual behavior and associated factors in Ethiopia: Further spatial and mixed effect analysis of Ethiopian Demographic Health Survey.***

**Manuscript Number**: PONE-D-23-21383

**General comments:**

The manuscript is well written, and the abstract, introduction, and other sections are clearly stated. The discussion and conclusions are in line with the key results. However**,** there are minor comments to be corrected, as suggested specifically below. The source of data for this study was 2016 DHS data, which is outdated, so there may have been many changes since then, and the findings might not show the recent events. The page numbers are not given.

**Abstract**:

1. Methods: On line 62, add the type of study design

**Introduction**:

2. On line 118, the authors did not include citations for previous studies conducted in Ethiopia. Please include a citation for existing evidence.
3. On lines 122-130, the authors did not elaborate well about the importance of exploring spatial analysis. What are the advantages of spatial analysis over other studies? Clearly describe the gaps and try to illustrate how the spatial analysis fulfills those gaps.

**Methods and Materials:**

4. On line 149, the study population is not clearly described.
5. The authors did not include the sub-sections in this manuscript. Sub-sections of eligibility criteria and data collection tools and procedures were missed. Please add these subsections in the methods part of the appropriate place.
6. The source of the data for this study was not also mentioned in the manuscript. Please describe the source of the data and put it in the proper place.
7. On line 152, what is the importance of sample weighting for DHS data analysis?
8. On lines 167 and 168, the authors did not put the operational definitions for community-level literacy and community-level poverty. However, you did not get these variables from the DHS data set; rather, they are composite variables developed from individual-level factors such as literacy status and wealth index. The problem of using these composite variables as level two without any evidence is multicollinearity. Repeated or twice-use of the same variables is unscientific. Therefore, I would like to recommend using only place of residence and region in the level two lists of variables.
9. On line 173, before spatial autocorrelation, please write spatial analysis as a sub-heading in the analysis. Then you can conduct step-by-step analysis: autocorrelation, hotspot analysis, interpolation, etc.
10. On line 210, why did you prefer to use LLR rather than deviance or AIC for model comparison? Which one is best for fitting multilevel binary logistic regression?
11. Data analysis: on line 234, what is the importance of fitting spatial regression using OLS in addition to fitting multilevel binary logistic regression? You can do spatial analysis with multilevel analysis or spatial analysis with spatial regression. Please choose either of the two.
12. After performing the OLS regression, the authors have reported that using GWR has poor model performance compared with the OLS output by using AIC and adjusted R square. The important question here is: Have you checked the important assumptions during the OLS regression analysis before comparing the model performance? The authors did not report the presence or absence of violations of important assumptions to choose either OLS regression or GWR. If you are interested in fitting spatial regression, please make assumptions. If the assumptions are violated, the model performance in the GWR is usually better than the OLS model. One important thing here is to not forget that this section is the methods part. Please don’t mix the result statements in the methods part. We cannot talk about model performance before data analysis; please rewrite this part as well. On the other hand, if you are interested in reporting spatial regression, please revise your title and objective in order to incorporate this.
13. On lines 243 and 244, this is one of the methods section parts, but the authors have written it as a result section. In the result section, you have reported that RSB was clustered across EAs with Moran’s index close to one (Moran’s I value of 0.82 (P-value <0.001). Moreover, your ICC report was 25%, which showed spatial heterogeneity between clusters. However, in the spatial regression analysis, you reported that there was no spatial heterogeneity of explanatory variables in RSB. Therefore, I have doubts that the spatial regression analysis was not conducted properly. I expect the presence of spatial non-stationarity. Therefore, I would like to recommend you re-analyze the spatial regression or report only spatial and multilevel analysis. However, the authors did not report OLS and GWR regression in the result section.

**Results**:

14. On lines 261 and 262, put the frequency and percentage figure with your text description.
15. On line 274, before sub-section' spatial autocorrelation', write first spatial analysis.
16. On lines 286-287: In the hotspot analysis output, why did you not report significant cold spots?
17. In table 2, what parameter did you use for model comparison, and which model was selected by what criteria?
18. According to your model analysis plan in the methods section, you did not perform the spatial regression analysis using OLS and GWR and you did not report the results the results section. Therefore, it is better to omit the paragraph statements in the methods section

**Discussion**:

19. On lines 372 to 381, please describe findings rather than directly results.
20. On lines 283-284, 397, and 409, please omit the AOR and 95% CI. The effect size is just enough.
21. At the end of the discussion, please add the strengths and limitations of your study.

**Conclusion**

22. On line 432, please replace the heading of the conclusion with “Conclusion and recommendation.
